# Ensuring continuous TB treatment across Asian borders

**DOI:** 10.5588/pha.23.0052

**Published:** 2024-03-01

**Authors:** A. Ohkado, S. Lee, A. Yoshie, K. Sugiura, S. Kasuya, K. Uchimura, A. Querri, A. P. Nguyen, I. A. Prayogi, K. Doi, L. Kawatsu

**Affiliations:** ^1^ Department of Epidemiology and Clinical Research, Centre for Japan Pre-Entry Tuberculosis Screening (JPETS) Quality Assessment, Research Institute of Tuberculosis (RIT), Japan Anti-Tuberculosis Association (JATA), Kiyose,; ^2^ Department of Respiratory Medicine, National Center for Global Health and Medicine, Shinjuku,; ^3^ Bridge TB Care, Department of Epidemiology and Clinical Research, RIT, JATA,; ^4^ School of Nursing, Nagoya City University, Nagoya, Japan

**Keywords:** migrants, tuberculosis patient care, referral, treatment outcome, loss to follow-up

## Abstract

**BACKGROUND:**

Mid-treatment cross-border migration of patients with TB increases the risk of treatment interruption.

**OBJECTIVE:**

To establish a cross-border referral process for patients with TB in Japan, and enhance their access to health facilities and treatment outcomes.

**DESIGN:**

This prospective cohort study describes and assesses the process of foreign-born patients with TB who returned to their home countries during treatment, focusing on their access to healthcare facilities and treatment outcomes.

**RESULTS:**

We enrolled 135 foreign-born patients with TB, and confirmed that 112 (83.0%) were referred to and accessed healthcare facilities after returning to their home countries. Of 102 patients due to complete treatment as of July 2023, 87 (85.3%) completed their treatment. We did not identify significant differences in the treatment success rate among patient characteristics, except between the patients with confirmed access to a healthcare facility and those without (*P* < 0.001). We confirmed that 49/87 (56.3%) patients had completed treatment with official data.

**CONCLUSION:**

The access and treatment success rates of the cross-bordered patients with TB from Japan were >80%; however, we should further improve this proportion by confirming the treatment outcomes with official data.

## Abstract (fr)

**CONTEXTE:**

La migration transfrontalière en milieu de traitement des patients atteints de TB augmente le risque d'interruption du traitement.

**OBJECTIF:**

Etablir un processus d'orientation transfrontalière pour les patients atteints de TB au Japon et à améliorer leur accès aux établissements de santé et les résultats de leur traitement.

**CONCEPTION:**

Cette étude de cohorte prospective décrit et évalue le processus des patients atteints de TB et nés à l'étranger qui sont retournés dans leur pays d'origine pendant le traitement, en se concentrant sur leur accès aux établissements de santé et sur les résultats du traitement.

**RÉSULTATS:**

Nous avons recruté 135 patients atteints de TB et nés à l'étranger et confirmé que 112 (83,0%) ont été orientés vers des établissements de santé et y ont accédé après leur retour dans leur pays d'origine. Des 102 patients qui devaient terminer leur traitement en juillet 2023, 87 (85,3%) l'ont terminé. Nous n'avons pas identifié de différences significatives dans le taux de réussite du traitement en fonction des caractéristiques des patients, sauf entre les patients ayant un accès confirmé à un établissement de santé et ceux qui n'en ont pas (*P* < 0,001). Nous avons confirmé que 49 (56,3%) des 87 patients avaient terminé leur traitement à l'aide des données officielles.

**CONCLUSION:**

Les taux d'accès et de réussite du traitement des patients transfrontaliers atteints de TB en provenance du Japon étaient >85% ; cependant, nous devrions encore améliorer cette proportion en confirmant les résultats du traitement à l'aide de données officielles.

TB remains one of the largest health burdens worldwide, with more than 10 million active cases and 1.6 million deaths per year.^1^ The global TB control target of a 90% treatment success rate set by the WHO, essential to contain TB burden worldwide, has not been achieved, and assisting all patients with TB in completing treatment is thus of paramount importance.^2^ Cross-border migration of patients with TB increases the risk of treatment interruption, with unfavourable treatment outcomes and potentially increasing the risk of acquired drug resistance,^3^ prolonged illness or death, and further transmission. The WHO and other international organisations have proposed the formalisation of a cross-border patient referral mechanism and systematic follow-up strategy;^4,5^ however, apart from the United States, a cross-border referral mechanism with data collection and analysis is currently ad hoc globally.^6–8^

Japan has a low incidence of TB (<10 per 100,000 population in 2021).^9^ The average proportion of foreign-born patients with TB reported in Japan was 11.8% in 2021, but substantially higher in the 15–34 years age group (over 60%).^9^ Approximately 5% of foreign-born patients with TB left Japan during treatment,^10^ including patients with multidrug-resistant TB (MDR-TB), whose subsequent access to healthcare services and treatment completion status is unknown.^11^ As Japan has not formalised a cross-border referral mechanism for patients with TB, a collection and analysis system for cross-border referral data does not exist. Generally, the physician in charge of a patient with TB who intends to cross the border writes a medical referral letter as a part of standard patient referral care, without further communication with the patient or National Tuberculosis Control Programme (NTP) staff in the destination countries. It is therefore challenging to determine the outcome for the patient after crossing the border.

This study aimed to describe the results of the Bridge TB Care (BTBC) Initiative of the Research Institute of Tuberculosis (RIT) in Tokyo, Japan, to ensure access to healthcare services and enable sharing of the treatment outcome of patients with TB who are transferred abroad.

## STUDY POPULATION, DESIGN AND METHODS

This prospective cohort study examined the issue of access to healthcare facilities and treatment outcomes for foreign-born patients with TB who left Japan for their home countries during treatment. The cross-border referral strategy for patients with TB, Bridge TB Care (BTBC), is shown in Supplementary Figure S1. The inclusion criteria of this study were as follows: all patients with TB (drug-susceptible and drug-resistant TB) who were born in countries such as the Philippines, China, Vietnam, Myanmar, Nepal and Indonesia, those who were diagnosed with active TB from August 2019 to July 2023 in Japan, those who were willing or decided to return to their home countries during treatment, and those who provided informed consent. We included these people as a cohort group and observed them towards the end of the TB treatment course. Public health centres that provided care to patients with TB in Japan initially contacted the RIT, Tokyo, using phone or email, and subsequently, sent a patient referral form (Supplementary Figure S2) and informed consent form to the RIT. Subsequently, the RIT contacted the relevant NTP staff member in the destination country to identify a suitable health facility to be accessed upon return. The engaged NTP staff consulted relevant administrative bodies, such as regional, provincial or city health offices, to identify an appropriate health centre or medical facility for the patient. In collaboration with local assistants assigned by the RIT, RIT staff collected information about the patient’s healthcare access and treatment outcomes. The RIT staff and the local assistants communicated with patients with TB via social networking services (SNS) such as ‘Facebook Messenger’ and ‘LINE’ so that they could assist patients and monitor their treatment status in their home countries. Before their departure, the RIT staff informed the patient through the Japanese health centre staff of the medical facility in the patient's home country. Then, with support from local assistants, the RIT staff communicated with the NTP staff regarding the patient’s attendance and treatment outcomes.

**FIGURE 1.**
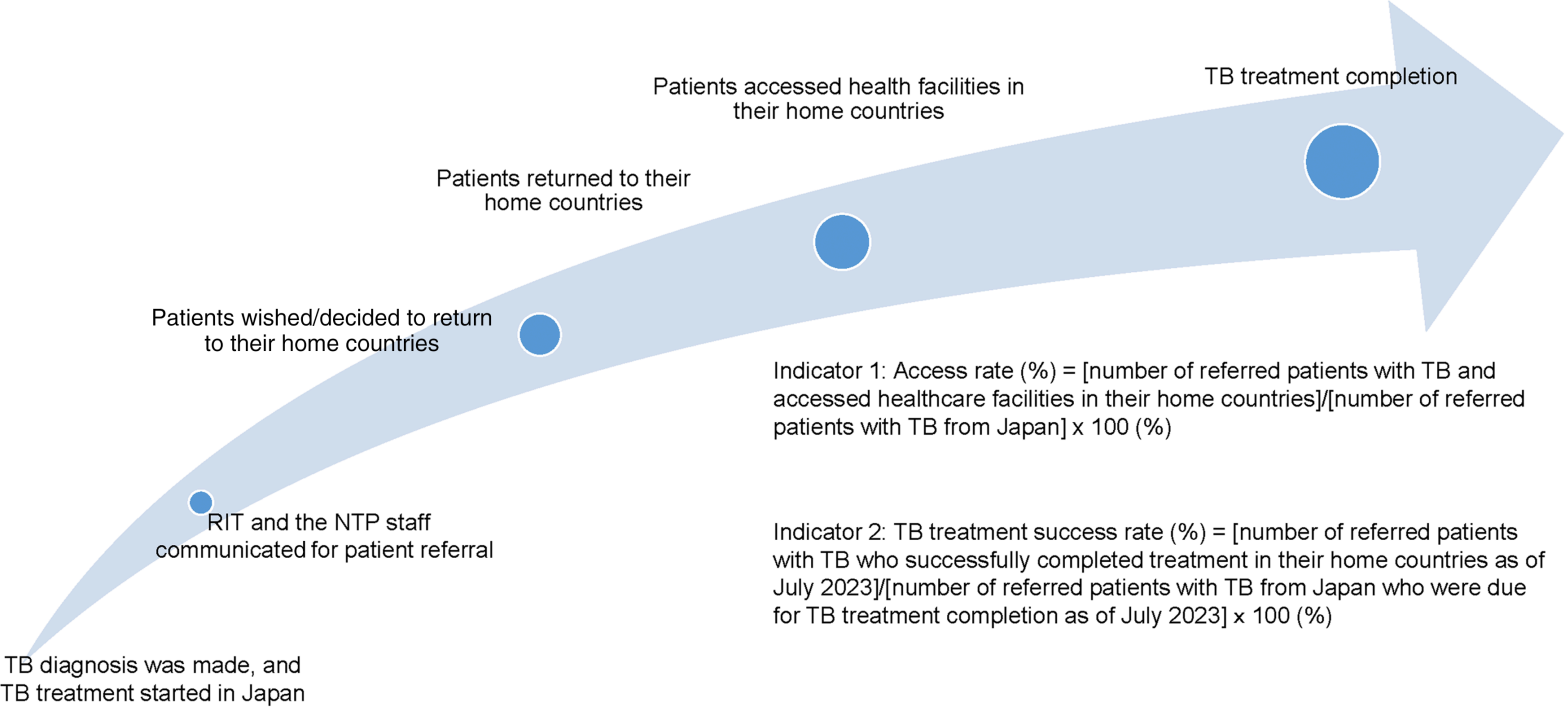
A pathway of foreign-born patients with TB crossing the border with Japan. RIT = Research Institute of Tuberculosis, Japan; NTP = National Tuberculosis Control Programme.

We quantified two major indicators for patients with TB in this programme: 1) access rate (AR) = [number of referred patients with TB who accessed healthcare facilities in their home countries]/[number of referred patients with TB from Japan] × 100 (%); and 2) TB treatment success rate (SR) = [number of referred patients with TB who successfully completed treatment in their home countries as of July 2023]/[number of referred patients with TB from Japan who were due for TB treatment completion as of July 2023] × 100 (%). In this analysis, TB treatment success was defined as ‘the TB treatment outcome of TB cases registered in the study period that completed treatment, whether as cured or treatment completed as of July 2023’. The information related to the two indicators has been obtained through official contact and informal contact with the patients and their relatives. Loss to follow-up was determined when the BTBC staff lost communication with TB cases referred for ≥2 months from the last contact. The pathway by which foreign-born patients with TB crossed the Japanese border is shown in Figure 1.

### Statistical analyses

We applied the χ^2^ or two-sided Fisher’s exact test to evaluate statistically significant differences between categorical data. We employed the Bonferroni correction for testing three pairs of categorical data, setting a statistically significant *P*-value at less than 0.017; for other tests, *P* < 0.05 was considered significant.

### Ethical considerations

We obtained informed consent from all patients with TB referred through the BTBC. The Institutional Review Board of the RIT, Japan, approved the study protocol (RIT/IRB 2019-06), and the study was completed in accordance with the Declaration of Helsinki.

## RESULTS

### Participant characteristics

**FIGURE 2.**
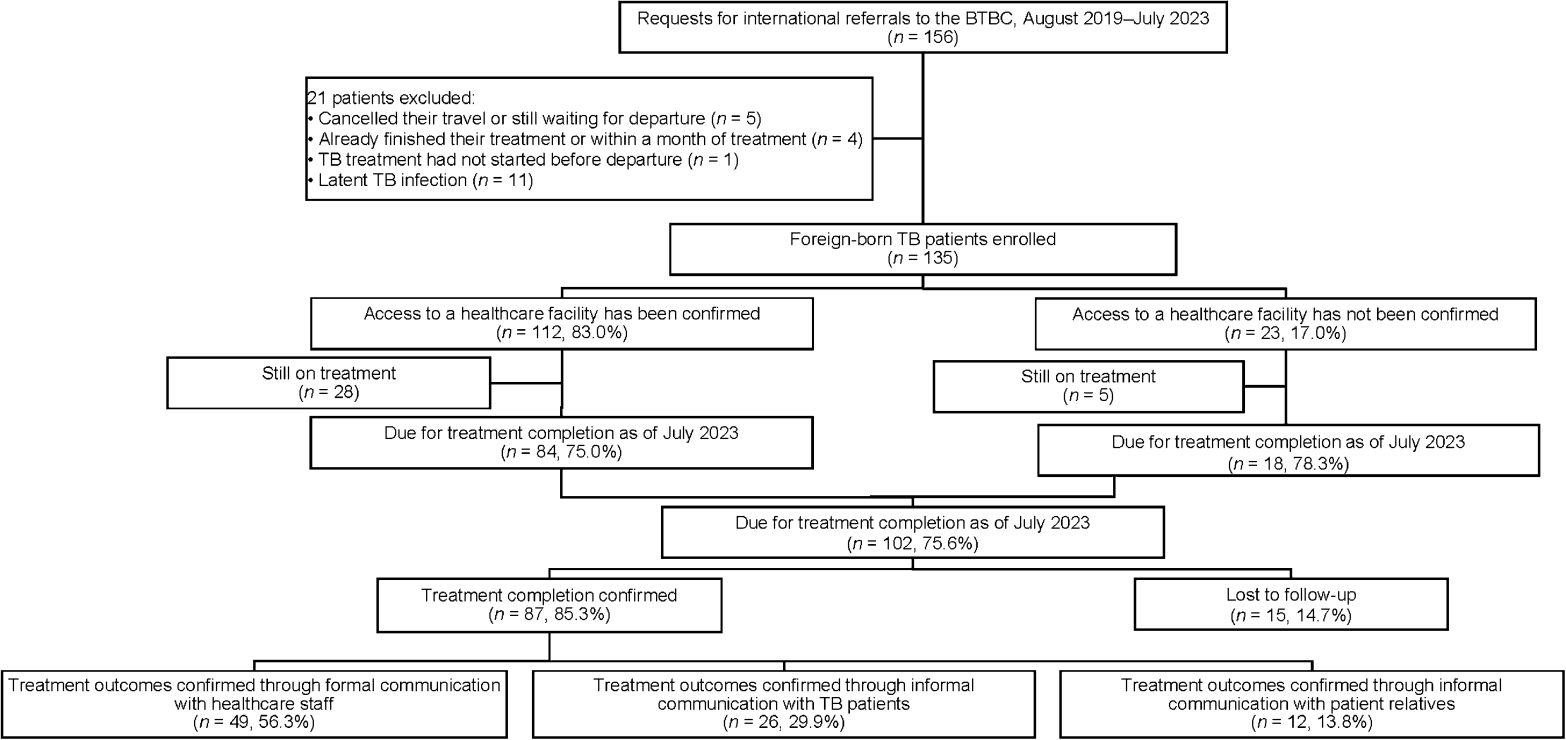
The enrolment flow of foreign-born patients with TB referred to the BTBC in Japan, September 2019–July 2023. BTBC = Bridge TB Care (a cross-border TB patient follow-up strategy in Japan).

As of July 2023, the BTBC had received requests for cross-border referrals of 156 patients with TB. After excluding those who had cancelled their travel, were still waiting for departure, had already finished their treatment before departure or had latent TB infections, we enrolled 135 foreign-born patients with TB from 11 countries, including 14 patients with rifampicin- or multidrug-resistant TB (RR/MDR-TB) (Figure 2). The Table shows the basic characteristics of the 135 foreign-born patients with TB enrolled in the study.

**TABLE.** Basic characteristics of the 135 foreign-born patients with TB enrolled in Japan, September 2019–July 2023.

	*n* (%)
Age, years, median [IQR]	27 [23–36]
Age category	
≤25	50 (37.0)
>25	85 (63.0)
Sex	
Males	81 (60.0)
Females	54 (40.0)
Destination countries	
Vietnam	35 (25.9)
The Philippines	28 (20.7)
Indonesia	26 (19.3)
China	16 (11.9)
Myanmar	7 (5.2)
Nepal	6 (4.4)
India	5 (3.7)
Other	12 (8.9)
Classification of TB patients by treatment history	
New	125 (92.6)
Retreatment	8 (5.9)
Unknown	2 (1.5)
Classification of TB patients by disease location	
Pulmonary TB	106 (78.5)
Extrapulmonary TB	29 (21.5)
Bacteriologically positive TB patients	104 (77.0)
Drug susceptibility test results	
Pan-susceptible TB patients*	53 (39.3)
RR/MDR-TB patients^†^	14 (10.4)
Drug-resistant TB patients other than RR/MDR-TB^†^	25 (18.5)
Not available	43 (31.9)
Chest X-ray findings	
With cavitary lesions	43 (31.9)
Without cavitary lesions	86 (63.7)
Unknown	6 (4.4)

* TB patients suffering from *Mycobacterium tuberculosis* susceptible to all of the first-line anti-TB drugs: isoniazid, rifampicin, ethambutol and streptomycin.

† TB patient suffered from rifampicin mono-resistant or rifampicin with isoniazid-resistant *Mycobacterium tuberculosis*.

IQR = interquartile range; RR/MDR-TB = rifampicin/multidrug-resistant TB.

### Access rate and TB treatment success rate

We confirmed that 112 of the 135 patients enrolled were successfully referred to a healthcare facility after returning to their home countries; the AR was 83.0%. Fifteen of the 23 patients with TB (65.2%) whose access to a healthcare facility could not be confirmed were subsequently lost to follow-up; 102 patients of the 135 patients were due for treatment completion as of the end of July 2023. Of these, we were able to confirm that 87 patients had successfully completed their TB treatment; the SR was 85.3%. There were no cases of TB treatment failure, unknown treatment outcomes or deaths. Of the 87 patients, 49 (56.3%) had their treatment outcomes confirmed using official information or clinical report, such as that provided by the NTP or medical facility staff. In the remaining patients, treatment outcomes were confirmed based on patient self-reports (29.9%) or reports by relatives (13.8%) (Figure 2).

**FIGURE 3.**
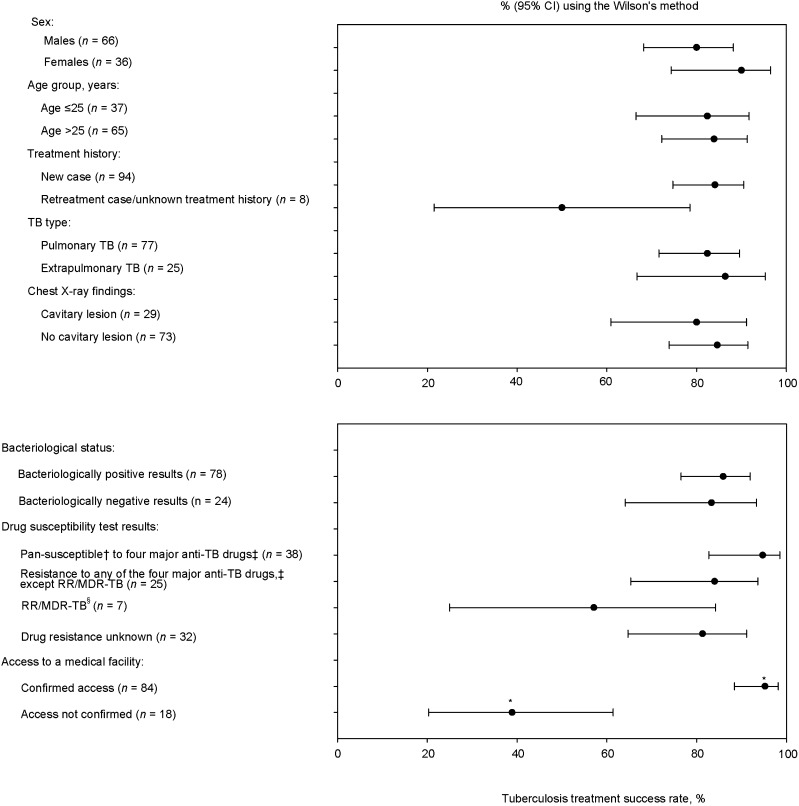
TB treatment success rate among the patients referred from Japan to home countries and due for treatment completion by 31 July 2023 by patient characteristic, 2019–2023 (*n* = 102;). *P < 0.0001. ^†^Pan-susceptible TB was defined as *Mycobacterium tuberculosis* susceptible to all of the first-line anti-TB drugs. ^‡^Isoniazid, rifampicin, ethambutol and streptomycin. ^§^RR/MDR-TB was defined as rifampicin mono-resistant or rifampicin with isoniazid-resistant *Mycobacterium tuberculosis* TB. RR/MDR-TB = rifampicin/multidrug-resistant TB.

No statistical differences in the SRs were observed among the home countries to which the patients returned (Supplementary Figure S3) or among different patient characteristics, except between the group with confirmed access to a medical facility and those without (*P* < 0.001; Figure 3A, 3B). Patients with RR/MDR-TB had a higher risk of being lost to follow-up than those with pan-susceptible test results, although this difference was not statistically significant (*P* = 0.021).

## DISCUSSION

This is the first study on the AR and SR among foreign-born patients with TB who left Japan during TB treatment and were systematically followed until treatment completion. This study showed that the BTBC achieved more than 80% in both AR and SR. In 2016, the WHO Western Pacific Region Office published a report on TB control in the migrant population to guide the NTPs in the region and facilitate NTP care in cross-border patients with TB.^12^ However, no systematic strategy has yet been established in the region to assist cross-border patients with TB in continuing their treatment. The present study showed the successful implementation of such a strategy, with an AR of 83.0% (112/135) and an SR of 85.3% (87/102). However, we could not confirm medical facility access for 23 foreign-born patients with TB; to note, 65.2% of these patients were subsequently lost to follow-up. It is thus imperative to confirm access to a medical facility in the destination country for cross-border patients with TB to ensure continuity of treatment.

We observed a substantial reduction in the number of foreign-born patients with TB notified in Japan, probably due to the COVID-19 pandemic that occurred during the present study period: a 16.4% reduction, from 1,541 patients in 2019 to 1,241 patients in 2022. The reduction in the number of foreign-born patients with TB enrolled in the study, from 34 patients in 2020 to 24 patients in 2021, was likely due to the reduction in the number of cross-bordering patients with TB. Nevertheless, the AR and SR were not similarly affected in the present study.

The SR in the present study was comparable to that reported by the US Centers for Disease Control and Prevention, in which 78% of patients (343/440) were referred by the San Diego County TB Control Branch, TB Cure, between 2012 and 2015.^13^ Furthermore, the Migrant Clinicians Network (TB Net) reported that 383 of 452 TB patients referred internationally (84.7%) completed their anti-TB treatment.^14^ The SR in the present study stratified by destination country did not present significant differences (Supplementary Figure S3), as the SR among Latin American countries was not provided in the TB Net report, which indicated that one in two patients with MDR-TB were lost to follow-up,^14^ and in a study in those who returned to the Philippines, only three out of five patients with MDR-TB completed TB treatment.^11^ It should be noted that as anti-TB treatment for patients with RR/MDR-TB is lengthy and tends to cause multiple side effects, treatment outcomes are likely to be poor. Understandably, the patients with RR/MDR-TB who move across borders during treatment encounter additional barriers to continuing anti-TB treatment after returning to home countries. The Cure TB report asserted that conducting interviews before patients left the United States was essential to ensure the continuity of anti-TB treatment in destination countries.^13^ The anti-TB drug regimens currently available for RR/MDR-TB treatment are not universally standardised, although the WHO has published guidelines on this topic.^15^ Therefore, more time is spent adjusting anti-TB drug regimens for cross-bordering patients with RR/MDR-TB than those with pan-susceptible TB. The close communication between medical staff within and outside of the country and patients with TB before they leave is critical, especially for those with RR/MDR-TB.

Local health staff officially confirmed that 56.3% of patients with TB crossed borders and completed anti-TB treatment. TB treatment outcomes without verification by health staff may be unreliable, as patients tend to claim that their adherence is good. We suggest enhancing the method for confirming TB treatment outcomes to attain fully official or clinical verification.

The referral of patients with TB does not ensure the continuity of patient care throughout TB diagnosis and treatment. Several studies reported that patients diagnosed with TB at public or private clinics were lost to follow-up after being referred domestically before initiating treatment.^16–18^ The referral of cross-border patients therefore requires rigorous care to ensure that patients can continue anti-TB medications without interruption. The BTBC maintained close communication with health centre staff in Japan and referred patients via email and/or SNS to assist patients in continuing treatment until completion. Furthermore, we assigned local assistants in four major receiving countries – the Philippines, Vietnam, Indonesia and China, who speak local languages other than English to communicate directly with patients and caregivers; we deemed it crucial for the BTBC intervention to have local assistants to maintain effective communication with patients throughout their care.

The present study had some limitations. First, there was no comparison group to assess the effectiveness of the BTBC intervention on the cross-border patients with TB, as the creation of a control group would have been impractical and unethical. Second, the referred patients with TB were selected by health centre staff after obtaining informed consent to allow the sharing of individual information with us. This may have resulted in a selection bias, as those who tended to be more adherent to anti-TB treatment may have been enrolled over those who tended to be less adherent. We could not know how many foreign-born patients with TB refused to provide informed consent but none withdrew consent after enrolling in the study. However, this study’s relatively high AR and SR may also reflect the enthusiastic patient support provided by the health centre and medical facility staff in Japan and their home countries. Furthermore, one of the strengths of the present study was that we involved the NTP of each destination country and local assistants who closely communicated with cross-border patients with TB. We also employed SNS to communicate with patients across borders. This allowed us to communicate closely and on time throughout the process. Nevertheless, further investigation into the backgrounds of those lost to follow-up is warranted.

The present study suggests that a cross-border referral strategy, such as the BTBC strategy, could contribute to successful treatment outcomes among patients with TB crossing the borders from Japan. The RIT has decided to continue to assist cross-bordering patients with TB with continuing treatment by strengthening health education in order that patients can access a healthcare facility soon after their arrival in their destination countries. However, the NTP needs to strengthen the TB patient referral mechanism inside and outside the country to sustain the cross-border referral system. Considering the critical role of local assistance in the receiving countries, the NTP should at least assign staff to coordinate with all relevant stakeholders to monitor cross-border TB patient referrals.

## CONCLUSIONS

The BTBC exhibited over 80% in both AR and SR. Currently, few cross-border referral programmes for patients with TB operate globally. The findings of this study could contribute to building valuable evidence for such programmes to ensure the continuity of patient care across borders.
