# Supplementary material for: Ensuring continuous TB treatment across Asian borders
Source: Public Health Action. 2024 Mar 1;14(1):20–5. doi: 10.5588/pha.23.0052 (PMC11122705; doi:10.5588/pha.23.0052)

http://dx.doi.org/10.5588/pha.23.0052

Supplementary Figure S1 The cross-border referral strategy for patients with tuberculosis, Bridge TB Care (BTBC)

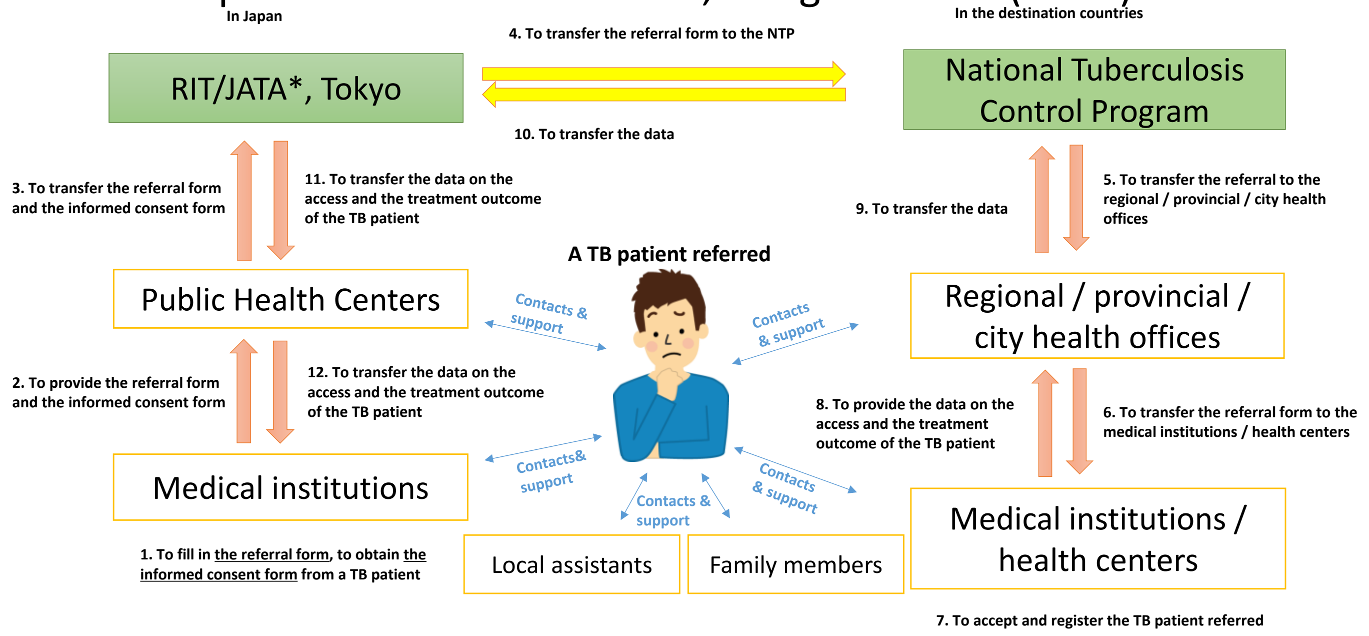

TB: tuberculosis

* RIT/JATA: Research Institute of Tuberculosis, Japan Anti-Tuberculosis Association, Tokyo, Japan

Supplementary Figure S2 Sample Referral Form for cross-border tuberculosis patient referral

	紹介ID Referral ID

結核患者紹介状 [海外転出者用] Tuberculosis Cross-border Referral (Page 1)

To be filled by the REFERRING country (Japan)

Referral phase I

照会元情報 Referrer information

受付日/First intake	day month year	結核登録者情報システム整理番号 /Registered ID	
登録保健所/Public Health Center		担当者/person in charge	
保健所電話番号/Telephone no		保健所Eメール/Email	
担当医/Name of referring physician:		紹介元医療機関/Referring medical facility	
担当医電話番号/Telephone no		担当医Eメール/Email	
医療機関住所/Address			

患者情報 Patient information

患者氏名/Name		性別/Sex		年齢/Age	
生年月日/Date of birth	day month year	Eメール/Email			
日本での電話番号/Phone no. in Japan		希望医療機関タイプ/Preference to type of medical facility			
日本での住所/Address in Japan					
その他の連絡先（名前）/Contact person in Japan (Name)		続柄/Relationship			
電話番号/Phone no		その他詳細/Specify if "Other" above			

臨床情報 Clinical information

診断名/Diagnosis		主な病巣部位/Site of TB		治療区分/Treatment history	

菌検査結果/Bacteriological status			感受性検査結果/drug susceptibility test results		胸部レントゲン/CXR	
			検査時期/Date (dd/mm/yyyy)	day month year		
	登録時/Initial result	直近/Most recent result	INH		空洞/Cavitary lesion	
検査時期/Date (dd/mm/yyyy)	day month year	day month year	RFP			
塗抹/smear			EB		その他所見/Other findings	
培養/culture			SM			
その他/others	Free text	Free text	その他の耐性 /Other resistance,	Free text	体重/Weight(kg)	kg

合併症/Co-morbidities	HIV		DM		その他/Others

治療状況/Medication				Remarks
抗結核薬/Anti-TB drugs	投与量/Dose	開始日/Start date	終了日/Stop date	Free text
INH	mg	day month year	day month year	
RFP	mg			
EB	mg			
SM	mg			
PZA	mg			
Others	Free text			

渡航先情報 Travel information

渡航予定日/Expected move date	day month year	渡航先/Destination country			
渡航先での電話番号/Phone no.		渡航先でのEmail/Email			
渡航先での住所/Address in destination country					
その他の連絡先（名前）/Contact person(Name)		続柄/Relationship		連絡可否/Agreemtnt to contact	
電話番号/Phone no		その他続柄詳細/Specify if "Other" above			

紹介ID Referral ID

Tuberculosis Cross-border Referral Form (Page 2)

To be filled by the RECEIVING country

Referral phase Ⅱ — 紹介先情報 — Administrative information

Date when referral form received	day month year		
Name of person in charge in the NTP		Email	
Affiliation			
Name of medical facility			
Name of receiving physician		Email	
Address of medical facility			
Date when referral form returned to JAPAN	day month year		
Date when referral form returned to PHC (or patient) in JAPAN	day month year		

Referral phase Ⅲ — 治療経過 — Follow-up

TB Registration ID in receiving country	ID code
Name of medical facility visited by patient	
Name of receiving physician	
Date of first vistit to medical facility	day month year
Date of TB registration	day month year
Date when treatment started	day month year

Treatment regimen when started

INH		Remarks
RFP		
EB		
SM		
PZA		
Others	Free text	

Referral phase Ⅳ — 治療成績 — Treatment outcome

Date when treatment completed	day month year
Treatment outcome (choose one of the options)	Comment
	Comments made by (name) ______ on day month year

To be filled by the REFERRING country (Japan)

Referral phase Ⅴ — Japan

Treatment outcome was received by (name) ______	on day month year
Treatment outcome was reported back to corresponding PHC by (name) ______	on day month year

This referral form was made by (name) ______ on day month year

BRIDGE TB CARE
結核医療国際連携支援

bridgetb@jata.or.jp
Department of Epidemiology and Clinical Research
The Research Institute of Tuberculosis
Japan Anti-Tuberculosis Association
3-1-24 MATSUYAMA, KIYOSE, TOKYO 204-8533, JAPAN
Phone: +81-42-493-5517
Fax: +81-42-493-5340
URL: http://www.jata.or.jp

ECR
Dept. of Epidemiology and Clinical Research
The Research Institute of Tuberculosis

Supplementary Figure S3

Figure S3 Tuberculosis treatment Success Rate of TB patients referred, from Japan to home countries from 2019 to 2023, by country referred to, patients due for treatment completion by July 31, 2023, n=102

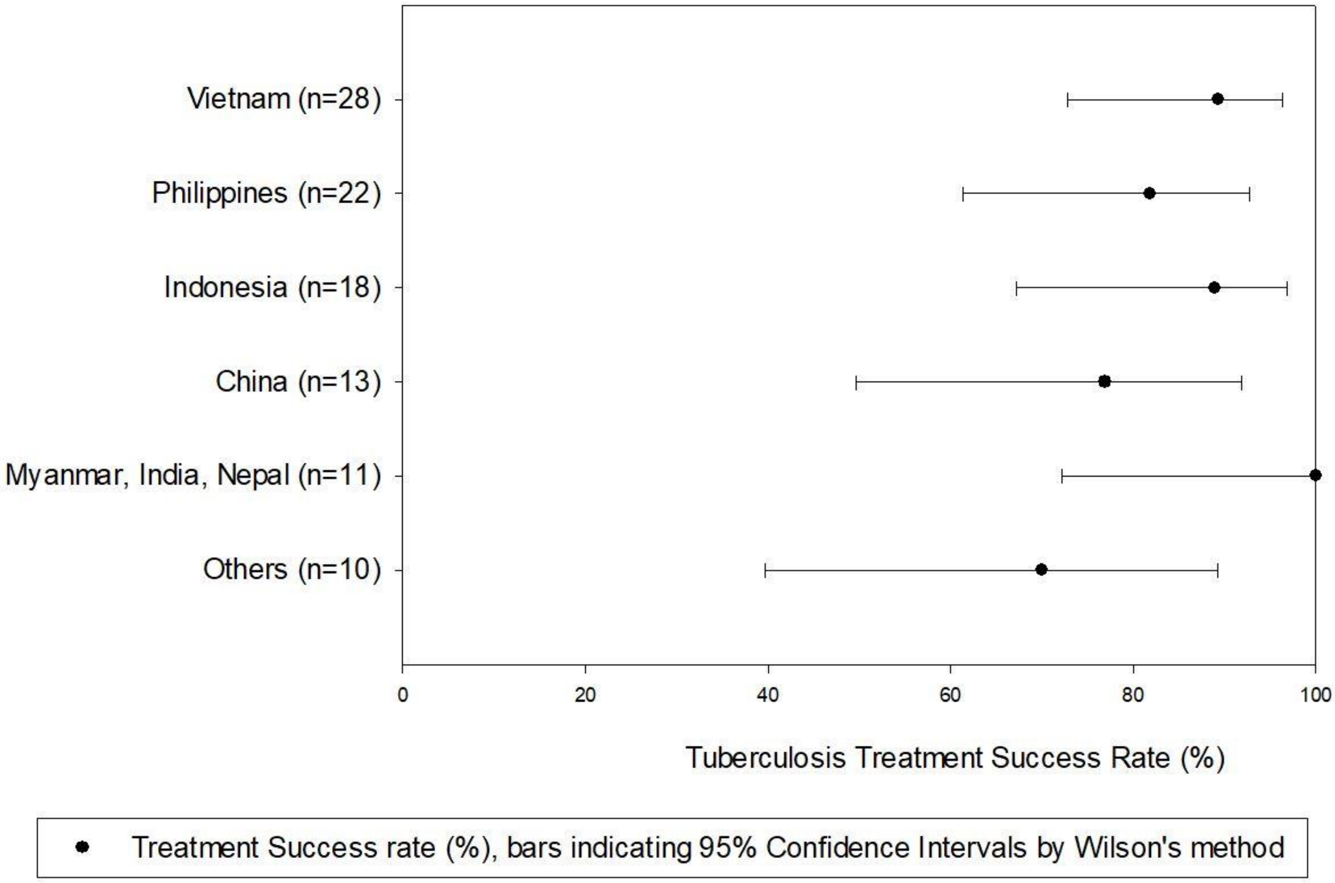

Supplement: Supplementary file 1 [file iutld_pha_23.0052_supplementarydata1.pdf]
